# Development and Validation of a New Simple Functional Score in the Older Chinese Population

**DOI:** 10.3389/fpubh.2022.813323

**Published:** 2022-02-24

**Authors:** Xingqi Cao, Chen Chen, Liu He, Zhoutao Zheng, Jingyun Zhang, Emiel O. Hoogendijk, Xiaoting Liu, Shujuan Li, Xiaofeng Wang, Yimin Zhu, Zuyun Liu

**Affiliations:** ^1^Center for Clinical Big Data and Analytics of the Second Affiliated Hospital and Department of Big Data in Health Science School of Public Health, Zhejiang University School of Medicine, Hangzhou, China; ^2^National Center for Acquired Immunodeficiency Syndrome/Sexually Transmitted Disease (AIDS/STD) Control and Prevention, Chinese Center for Disease Control and Prevention, Beijing, China; ^3^Department of Epidemiology and Data Science, Amsterdam Public Health Research Institute, Amsterdam UMC-Location VU University Medical Center, Amsterdam, Netherlands; ^4^School of Public Affairs, Zhejiang University, Hangzhou, China; ^5^Department of Neurology, Beijing Chaoyang Hospital, Capital Medical University, Beijing, China; ^6^National Clinical Research Center for Aging and Medicine, Huashan Hospital, and Human Phenome Institute, Fudan University, Shanghai, China; ^7^Department of Epidemiology and Biostatistics, School of Public Health, Zhejiang University School of Medicine, Hangzhou, China

**Keywords:** aging, Chinese, cognitive function, mortality, physical function

## Abstract

**Background:**

Existing aging metrics incorporating cognitive and physical function are often not feasible for application in research and clinical practice. Therefore, this study aimed to develop and validate a new simple functional score based on self-reported cognitive and physical function in the older Chinese population.

**Methods:**

The development sample included 3,929 older adults aged 60–95 years from the China Health and Retirement Longitudinal Study (CHARLS). The validation sample included 1,345 older adults aged 60–87 years from the Rugao Longitudinal Aging study (RLAS). Logistic regression models and receiver operating characteristic curves were used to examine the associations of the new functional score with all-cause mortality risk.

**Results:**

Six items were selected to construct the new functional score in CHARLS. This functional score was associated with all-cause mortality risk, with an adjusted odds ratio of 1.10 (95% confidence interval = 1.07, 1.13). This functional score presented additional predictive utility beyond age and sex, as demonstrated by the significantly increased C-statistic, integrated discrimination improvement (IDI), and continuous net reclassification improvement (NRI) (all *P* < 0.001). Furthermore, this functional score was further validated in RLAS, such that adding the new functional score to a model of age and sex improved all-cause mortality risk discrimination (IDI = 0.036, *P* < 0.001; NRI = 0.485, *P* < 0.001). To facilitate the quick screening of the older population with deteriorations in cognitive and physical function, we introduced a publicly available online tool designed for this new functional score.

**Conclusions:**

A new functional score based on six self-reported items was developed and validated in the older Chinese population, and was demonstrated to be a simple and practical tool to assess functional deterioration, showing good feasibility, and performance.

## Introduction

Aging is an irreversible and complex process of multi-system physiological dysregulation. To better assess the aging process, aging metrics incorporating biological or functional markers have been proposed. Functional metrics of aging usually comprise cognitive and physical function, reflecting individual health status in different domains of physiological function, and perform well in predicting downstream health outcomes including mortality (1, 2). Cognitive impairment ranges in severity from mild to severe due to the deteriorations in cognitive domains such as memory, learning, and/or executive function. Physical function refers to the capability to perform activities and is usually assessed by various subjective [e.g., basic activities of daily living [BADL] (3), instrumental activities of daily living [IADL] (4)] and objective [e.g., short physical performance battery [SPPB] (5), timed up and go test (6), grip strength (7), and gait speed (7)] tools. Physical frailty (PF) is a state of being vulnerable to stressor events due to the cumulative physiological declines in multiple systems (8, 9), contributing to the decline in physical function (10). PF usually affects mobility first, and further results in disability (8). Abundant evidence showed that the combined presence of cognitive impairment and PF contributes to a significantly increased risk of adverse outcomes (11–16).

There are at least three metrics that have incorporated cognitive and physical function in previous studies (17–19). The first one is cognitive frailty, which was defined as the coexistence of cognitive impairment and PF in non-dementia older populations, and proposed by an International Association of Gerontology and Geriatrics (IAGG) consensus group in 2013 (17). The second one is the frailty index (FI) that integrates cognitive and physical phenotypes into a single-dimensional index, reflecting cumulative health deficits (18). The third one is motoric cognitive risk syndrome (MCR), which was characterized by the concurrence of subjective cognitive complaints with slow gait speed in older adults without dementia and mobility disability, and proposed by Verghese et al. (19). Although these metrics are conceptually overlapping, they are different to some extent. More importantly, these metrics have some drawbacks. Measurements of both cognitive frailty and MCR require physical examination (e.g., measurement of gait speed), which is often not feasible in clinical practice. The FI comprises many items (≥30 items) and requires extensive data collections, which hampers its application in research and practice. In addition, the cognitive function has equal weight as physical function in cognitive frailty, FI, and MCR. This might not be true in the real world. Given these limitations aforementioned, there is a need to develop a new simple metric with practical value by incorporating cognitive and physical function simultaneously.

Therefore, this study first aimed to develop a new simple functional score by integrating self-reported cognitive and physical function items and examine the predictive utility for all-cause mortality, in the China Health and Retirement Longitudinal Study (CHARLS). Additionally, we validated this new functional score in the Rugao Longitudinal Aging Study (RLAS), an independent dataset. To further facilitate the quick screening of the older population with deteriorations in cognitive and physical function, we introduced a publicly available online tool designed for this new functional score.

## Materials and Methods

### Study Population

CHARLS is a nationally representative prospective cohort study of adults aged 45 years and above in China initiated in 2011/2012 and followed up every 2 years. As described elsewhere (20), the multistage probability proportional to size sampling strategy was adopted to identify participants through four stages according to their regions, urban or rural countries, and statistics on the gross domestic product. CHARLS has been approved by the Ethical Review Committee at Peking University and all participants provided written informed consent. The baseline survey (2011/2012) recruited 17,708 participants aged 45 years and older. Those who had disability in BADL (*N* = 1,462), or had the memory-related disease (*N* = 103), aged below 60 years (*N* = 10,124), or with missing data on demographic covariates (*N* = 6) and items for constructing aging metrics (*N* = 2,084) were excluded. Finally, 3,929 participants aged 60–95 years were included in this study.

RLAS is a community-based longitudinal study conducted in Rugao, Jiangsu Province, China (21). In 2014, RLAS recruited participants from 31 rural communities of Jiang'an Township, Rugao, according to 5-year age and sex strata. A total of 1,960 participants were recruited to complete questionnaires, physical examinations, and provided biological samples. The follow-up survey was conducted in 2016, 2017, and 2019 for repeated measurements of health status. Due to the data availability, we included 1,345 participants aged 60–87 years from the 2016 wave to validate the new functional score in this study. The Human Ethics Committee of the School of Life Science at Fudan University approved the RLAS. Written informed consent was obtained from all RLAS participants.

### All-Cause Mortality

The death information in CHARLS was collected at the exit interview of each survey during follow-up. But the exact date of death was not available in the 2015 and 2018 waves. Therefore, we defined a binary variable to denote the occurrence of death within the 6-year follow-up since baseline.

The death information in RLAS was collected from the Funeral home of Rugao and Rugao Civil Affairs Bureau. The village or community doctors were responsible to investigate and validate the cause of death.

### Covariates

Covariates in CHARLS including age, sex, residence, education, and disease count were collected at baseline. The residence was defined as urban or rural. Educational level was defined as illiterate, elementary school, middle school, high school, or college and higher than college. We counted the total number of chronic diseases (including hypertension, diabetes or high blood sugar, cancer or malignant tumor, chronic lung disease, heart problems, stroke, kidney disease, stomach or other digestive diseases, arthritis or rheumatism, and asthma), and then classified disease count into 5 categories: 0 disease, 1 disease, 2 diseases, 3 diseases, and 4 or more diseases. Additionally, we measured depression by the 10-item Center for the Epidemiological Studies of Depression Short Form (CESD-10) (20). The summary score of CESD-10 ranges from 0 to 30, with higher scores indicating severer depressive symptoms during the last week.

Covariates in RLAS including age, sex, education, and diseases count were collected in the 2016 wave. Education level was defined as illiterate or literate (≥1-year education). We counted the total number of chronic diseases (including hypertension, diabetes, cancer or malignant tumor, chronic lung disease, heart problems, stroke, kidney disease, stomach or other digestive diseases, arthritis or rheumatism, and asthma), and then classified disease count into 5 categories as done in CHARLS.

### Development of the New Functional Score

Building on the findings that cognitive frailty and FI performed relatively better in predicting all-cause mortality compared to another measure (22), we developed a new simple functional score that integrated cognitive frailty and FI. We ran a stepwise logistic regression model to identify candidate items from components of cognitive frailty and FI for predicting all-cause mortality. Then, the new score was calculated in four steps (23). First, we ran a multivariable logistic model that includes age, sex, education, and candidate items to estimate the effect of each item independent of potential confounders and other items. Second, we calculated the individual risk point for each item by dividing the corresponding regression coefficient with a single constant, which represents the regression coefficient for a 1-year increase in age with the risk of all-cause mortality. Third, we rounded the risk points to the nearest integers. Fourth, we calculated the composite score by summing the individual risk point for each candidate item of each participant. Considering that several self-reported diseases (e.g., chronic lung disease, heart disease) items were retained in the stepwise logistic regression models, we replaced them with one disease count variable. After carefully screening self-reported items for all-cause mortality prediction and their properties (e.g., reflect cognitive or physical function), we included one item for cognition and five items for physical function to develop the new functional score. Cognition was assessed by serial subtraction of 7 from 100 up to five times, with a score range from 0 to 5. Weight loss was defined as having a body mass index (BMI) of 18.5 kg/m^2^ or less, or a self-reported weight loss of 5 kg or more in the past year. Chronic diseases included ten self-reported conditions as mentioned above. The total number of chronic diseases was calculated. We classified disease count into 5 categories: 0 disease, 1 disease, 2 diseases, 3 diseases, and 4 or more diseases. Limitations in running/jogging, walking, and climbing stairs were measured by asking participants whether they have difficulty in running/jogging 1 km, have limitations in walking 1 km, and have limitations in climbing several flights of stairs, respectively. The detailed scores for each item were presented in Table 1. The summary score (i.e., the new functional score) ranged from 0 to 20, with the higher score indicating worse function. The estimation of all-cause mortality risk for the functional score was presented in Supplementary Table 1.

**Table 1:** Components of the new simple functional score in CHARLS.

**Components**	**Construction**		**Person #1**	
	**Category**	**Risk points**	**Response**	**Points**
Serial subtraction of 7 from 100	0	4	2	2
	1	3		
	[2, 3]	2		
	4	1		
	5	0		
Having a BMI of 18.5 kg/m^2^ or less	No	0	No	0
	Yes	5		
Disease count	[0, 1]	0	2	1
	[2, 3]	1		
	≥4	2		
Limitations in running/jogging 1 km	No	0	Yes	3
	Yes	3		
Limitations in walking 1 km	No	0	No	0
	Yes	5		
Limitations in climbing several flights of stairs	No	0	Yes	1
	Yes	1		
	Total points	0–20	Total points	7
			Estimate of risk	0.063

*CHARLS, China Health and Retirement Longitudinal Study; BMI, body mass index*.

### Statistical Analyses

All statistical analyses were performed using R version 3.6.3 (2020-02-29) and SAS version 9.4 (SAS Institute, Cary, NC). A *P* value of < 0.05 (two-tailed) was considered statistically significant. We described characteristics of participants using mean ± standard deviation (SD) for continuous variables or number (percentages) for categorical variables.

We used 3 logistic regression models to examine associations of the new functional score with all-cause mortality risk in CHARLS. The odds ratios and corresponding 95% confidence intervals (CIs) were calculated. Model 1 was a crude model. Model 2 adjusted for age and sex. Model 3 additionally adjusted for residence and education. ROC curves were then used to evaluate the predictive utility of the new functional score for all-cause mortality risk. We calculated the delta C-statistic, integrated discrimination improvement (IDI) (24), and continuous net reclassification improvement (NRI) (24) in comparison to that of the basic model with age and sex. Delta C-statistic equals to x% means that the difference in predicted risks between the persons with and without the outcome increased by x% in the updated model. IDI equals to x% means that the difference in average predicted risks between the persons with and without the outcome increased by x% in the updated model. Continuous NRI equals to x% means that compared with persons without outcome, persons with outcome were almost x% more likely to move up a category than down. With a given cut-off, NRI might be a better choice; otherwise, IDI may be preferred (24). Finally, we evaluated the associations of the new functional score with all-cause mortality in RLAS using the same analytic models above.

We performed several sensitivity analyses to test the robustness of our findings. First, to account for the influence of depression on the associations, we repeated the main analysis (i.e., testing the association of the new functional score with all-cause mortality risk) with additional adjustment for depression (assessed by CESD-10) based on Model 3 in CHARLS. Second, there were three existing metrics integrating cognitive and physical function in literature, and thus, we evaluated the predictive ability for all-cause mortality risk when adding the new functional score to a model including one existing metric (i.e., cognitive frailty, FI, or MCR), age, and sex in CHALRS.

## Results

The characteristics of the study participants in CHARLS are presented in Table 2. The mean age of the 3,929 participants in CHARLS was 67.4 (*SD* = 6.3) years. About 53.3% (*N* = 2,102) were males. The proportions of rural residence and illiteracy were 61.8% (*N* = 2,427) and 33.0% (*N* = 1,296), respectively. During 6 years of follow-up, 574 participants died (14.6%). In RLAS, the mean age of the 1,345 participants was 77.2 (*SD* = 3.9) years, and the proportion of males was 46.4% (Supplementary Table 2). During 3 years of follow-up, 135 participants died (10.0%).

**Table 2:** Summary characteristics of the study participants in CHARLS.

**Characteristics**	**Total**	**Male**	**Female**
*N*	3,929	2,102	1,827
Age, mean ± SD	67.4 ± 6.3	67.4 ± 6.1	67.4 ± 6.6
Male, *N* (%)	2,102 (53.5)	—	—
Residence, rural, *N* (%)	2,427 (61.8)	1,329 (63.2)	1,098 (60.1)
**Education**			
No schooling, *N* (%)	1,296 (33.0)	352 (16.8)	944 (51.7)
Primary school, *N* (%)	1,859 (47.3)	1,181 (56.2)	678 (37.1)
Middle school, *N* (%)	511 (13.0)	368 (17.5)	143 (7.8)
High school or more, *N* (%)	263 (6.7)	201 (9.6)	62 (3.4)
**Marital status**			
Currently married, *N* (%)	3,064 (78.0)	1,780 (84.7)	1,284 (70.3)
Others, *N* (%)	865 (22.0)	322 (15.3)	543 (29.7)
**Smoking status^a^**
Non-smoker, *N* (%)	2,639 (67.2)	963 (45.8)	1,676 (91.7)
Smoker, *N* (%)	1,289 (32.8)	1,138 (54.2)	151 (8.3)
**Alcohol consumption**			
Non-drinker, *N* (%)	2,291 (58.4)	746 (35.5)	1,545 (84.6)
Drinker, *N* (%)	1,635 (41.7)	1,354 (64.5)	281 (15.4)
BMI (kg/m^2^), mean ± SD	22.9 ± 3.9	22.4 ± 3.6	23.4 ± 4.1
**BMI category**			
Underweight, *N* (%)	397 (10.1)	209 (10.0)	188 (10.3)
Normal, *N* (%)	2,154 (55.0)	1,276 (60.9)	878 (48.1)
Overweight, *N* (%)	1,015 (25.9)	474 (22.6)	541 (29.6)
Obese, *N* (%)	354 (9.0)	136 (6.5)	218 (12.0)
**Disease count^b^**			
0, *N* (%)	1,116 (28.4)	633 (30.1)	483 (26.4)
1, *N* (%)	1,252 (31.9)	678 (32.3)	574 (31.4)
2, *N* (%)	885 (22.5)	437 (20.8)	448 (24.5)
3, *N* (%)	426 (10.8)	226 (10.8)	200 (11.0)
≥4, *N* (%)	250 (6.4)	128 (6.1)	122 (6.7)
CESD-10, mean ±*SD*	7.9 ± 5.9	7.1 ± 5.5	8.8 ± 6.2

*CHARLS, China Health and Retirement Longitudinal Study; SD, standard deviation; BMI, body mass index; CESD-10, 10-item Center for the Epidemiological Studies of Depression Short Form*.

a *Percentages may not sum to 100 because of rounding. There were 1 participant with missing data on smoking status, 3 participants with missing data on drinking status, 9 participants with missing data on BMI*.

a *In CHARLS, chronic diseases included hypertension, diabetes or high blood sugar, cancer or malignant tumor, chronic lung disease, heart problems, stroke, kidney disease, stomach or other digestive diseases, arthritis or rheumatism, and asthma*.

Table 3 presents the associations of the new functional score with all-cause mortality in CHARLS. In the crude model, a 1-score increase in the functional score increased the risk of all-cause mortality by 13% (*OR* = 1.13, 95% *CI* = 1.11, 1.16). The ORs for all-cause mortality in the second, third, fourth, and fifth quartile of the new functional score were 1.75 (95% *CI* = 1.27, 2.39), 1.83 (95% *CI* = 1.31, 2.57), 2.44 (95% *CI* = 1.79, 3.32), and 4.72 (95% *CI* = 3.48, 6.40), respectively, compared with that in the first quartile. After adjusting for demographic covariates, these results did not change substantially (models 2 and 3 in Table 3).

**Table 3:** Associations of the new functional score with all-cause mortality in CHARLS.

		**No. of events/No.** **of participants**	**Model 1**		**Model 2**		**Model 3**	
			**OR (95% CI)**	***P***	**OR (95% CI)**	***P***	**OR (95% CI)**	***P***
New functional score	Per 1 score	574/3,929	1.13 (1.11,1.16)	<0.001	1.11 (1.09, 1.14)	<0.001	1.10 (1.07, 1.13)	<0.001
Quintiles	Q1	67/894	Ref.	–	Ref.	–	Ref.	–
	Q2	117/944	1.75 (1.27, 2.39)	<0.001	1.60 (1.15, 2.21)	0.005	1.47 (1.06, 2.04)	0.021
	Q3	85/657	1.83 (1.31, 2.57)	<0.001	1.77 (1.24, 2.51)	0.002	1.64 (1.15, 2.34)	0.006
	Q4	135/819	2.44 (1.79, 3.32)	<0.001	2.25 (1.62, 3.13)	<0.001	1.96 (1.40, 2.74)	<0.001
	Q5	170/615	4.72 (3.48, 6.40)	<0.001	3.70 (2.65, 5.16)	<0.001	3.20 (2.28, 4.50)	<0.001
	P for trend		—	<0.001	—	<0.001	—	<0.001

*CHARLS, China Health and Retirement Longitudinal Study; OR, odds ratio; CI, confidence interval; Q1, the first quintile; Q2, the second quintile; Q3, the third quintile; Q4, the fourth quintile; Q5, the fifth quintile*.

Model 1 was a crude model.

Model 2 adjusted for age and sex.

Model 3 further adjusted for residence and education based on Model 2.

**Figure 1:**
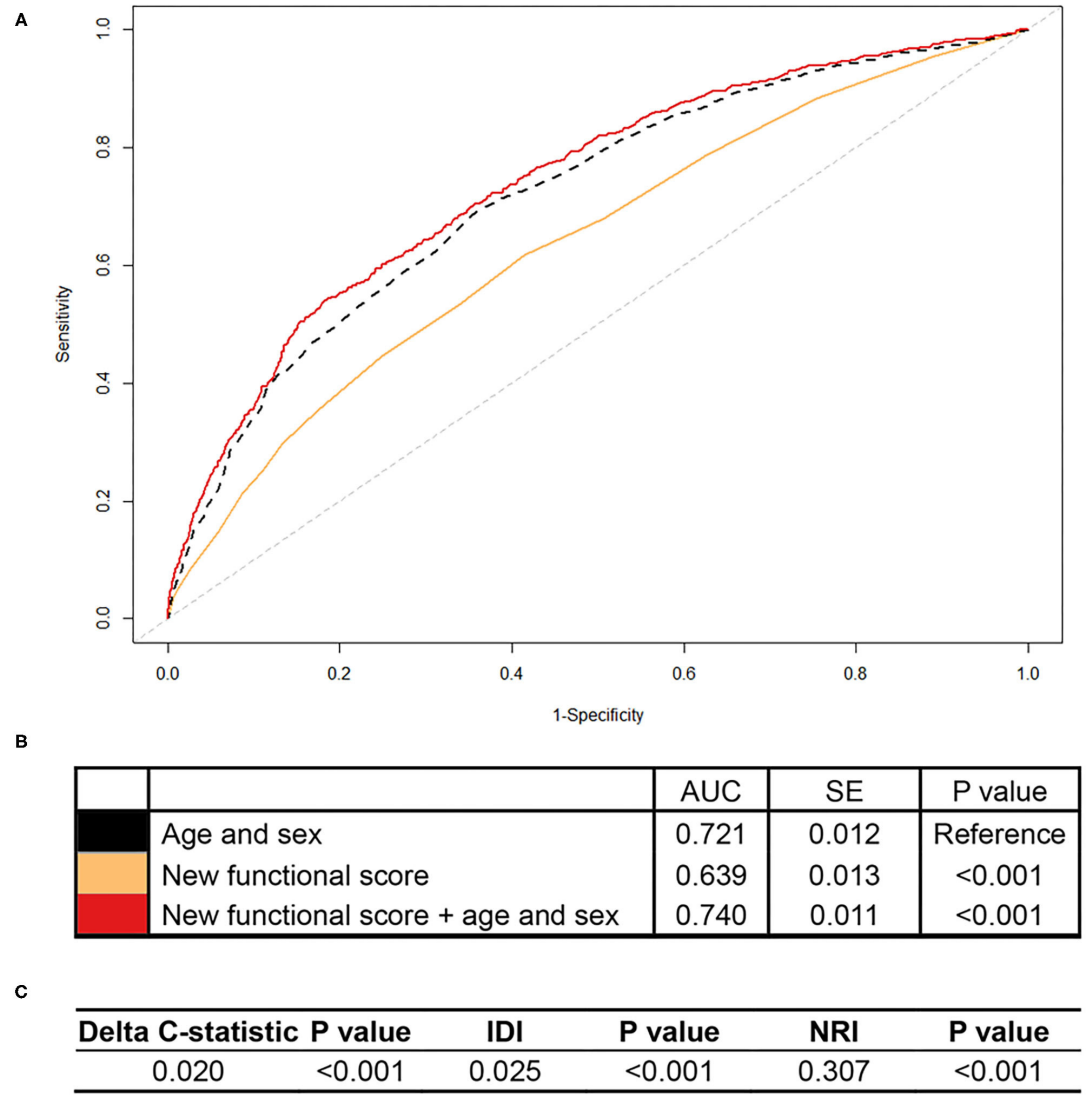
Association of the new functional score with all-cause mortality in CHARLS. CHARLS, China Health and Retirement Longitudinal Study; AUC, area under the curve; SE, standard error; IDI, integrated discrimination improvement; NRI, net reclassification index. We calculated the continuous NRI and IDI using R package “PredictABEL,” in comparison to that of the basic model with age and sex. NRI equals to x% means that compared with persons without outcome, persons with outcome were almost x% more likely to move up a category than down. IDI equals to x% means that the difference in average predicted risks between the persons with and without the outcome increased by x% in the updated model. **(A)** Shows receiver-operator characteristics curves for prediction of all-cause mortality for the new functional score. **(B)** Shows the AUC for each model. **(C)** Shows delta C-statistic, IDI, and NRI, in comparison to that of the basic model with age and sex.

As shown in Figure 1, the area under the curve (AUC) for all-cause mortality prediction by the new functional score was 0.639 in CHARLS. This new functional score added predictive utility to the basic model with age and sex only, with an AUC of 0.740, which was significantly higher than that of the basic model (i.e., 0.721). Additionally, the model including the new functional score had better discrimination and reclassification ability, as assessed by significantly increased delta C-statistic (i.e., 0.020), IDI (i.e., 0.025), and continuous NRI (i.e., 0.307).

**Figure 2:**
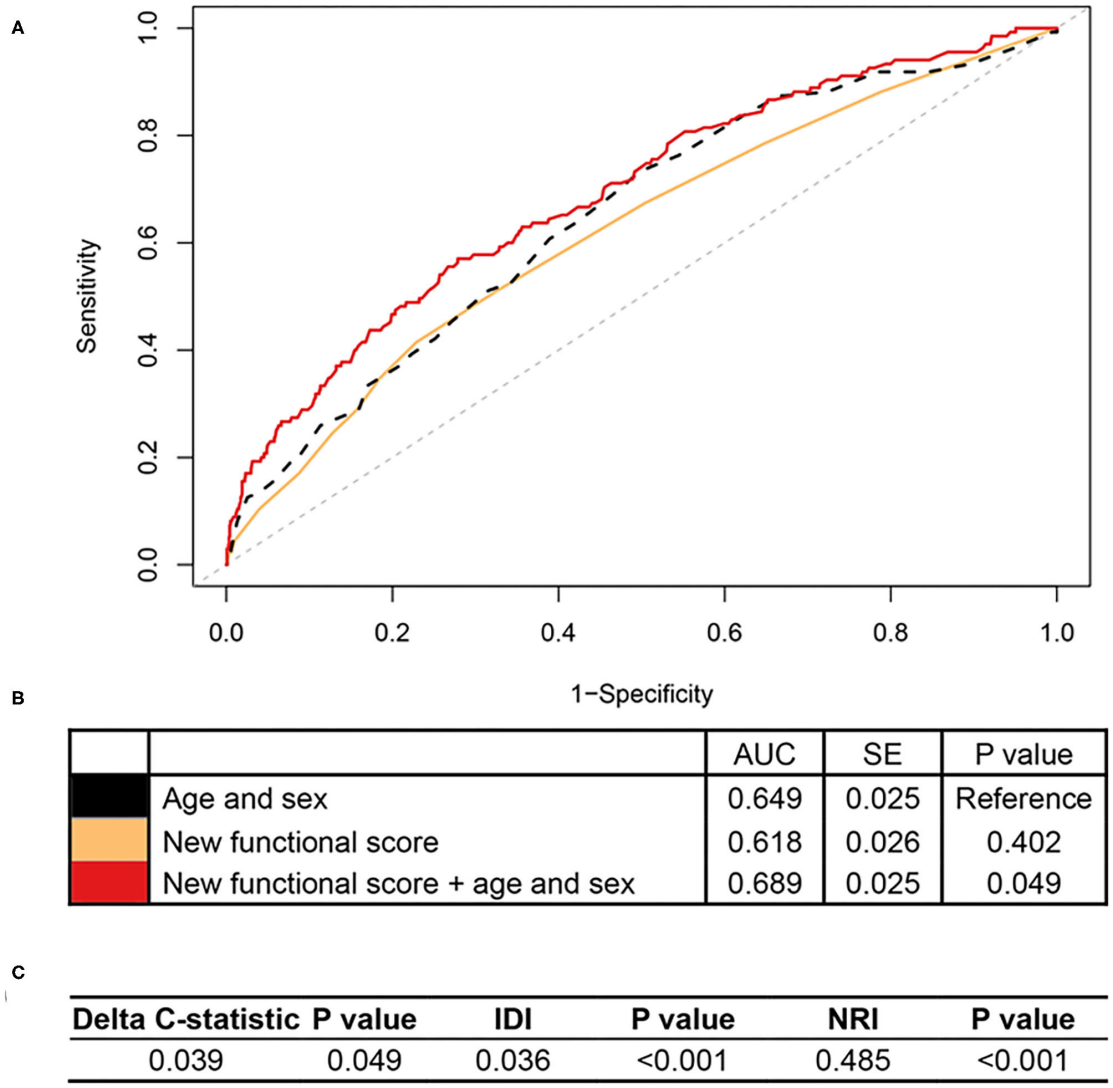
Association of the new functional score with all-cause mortality in RLAS. RLAS, Rugao Longitudinal Aging Study; AUC, area under the curve; SE, standard error; IDI, integrated discrimination improvement; NRI, net reclassification index. We calculated the continuous NRI and IDI using R package “PredictABEL,” in comparison to that of the basic model with age and sex. NRI equals to x% means that compared with persons without outcome, persons with outcome were almost x% more likely to move up a category than down. IDI equals to x% means that the difference in average predicted risks between the persons with and without the outcome increased by x% in the updated model. **(A)** Shows receiver-operator characteristics curves for prediction of all-cause mortality for the new functional score. **(B)** Shows the AUC for each model. **(C)** Shows delta C-statistic, IDI, and NRI, in comparison to that of the basic model with age and sex.

In RLAS, an independent dataset, we found that the new functional score predicted all-cause mortality as well, with an AUC of 0.618 (standard error = 0.026) (Figures 2A,B). More importantly, we found that the new functional score added predictive utility to the basic model with age and sex only. The AUC for mortality prediction was higher for a model with the new functional score, age, and sex (i.e., 0.689), relative to that of the basic model (i.e., 0.649). Adding the new functional score contributed significant improvements for predicting all-cause mortality in terms of reclassification, evidenced by the significant increase in IDI and continuous NRI relative to that of the basic model (all *P* < 0.05, Figure 2C).

**Figure 3:**
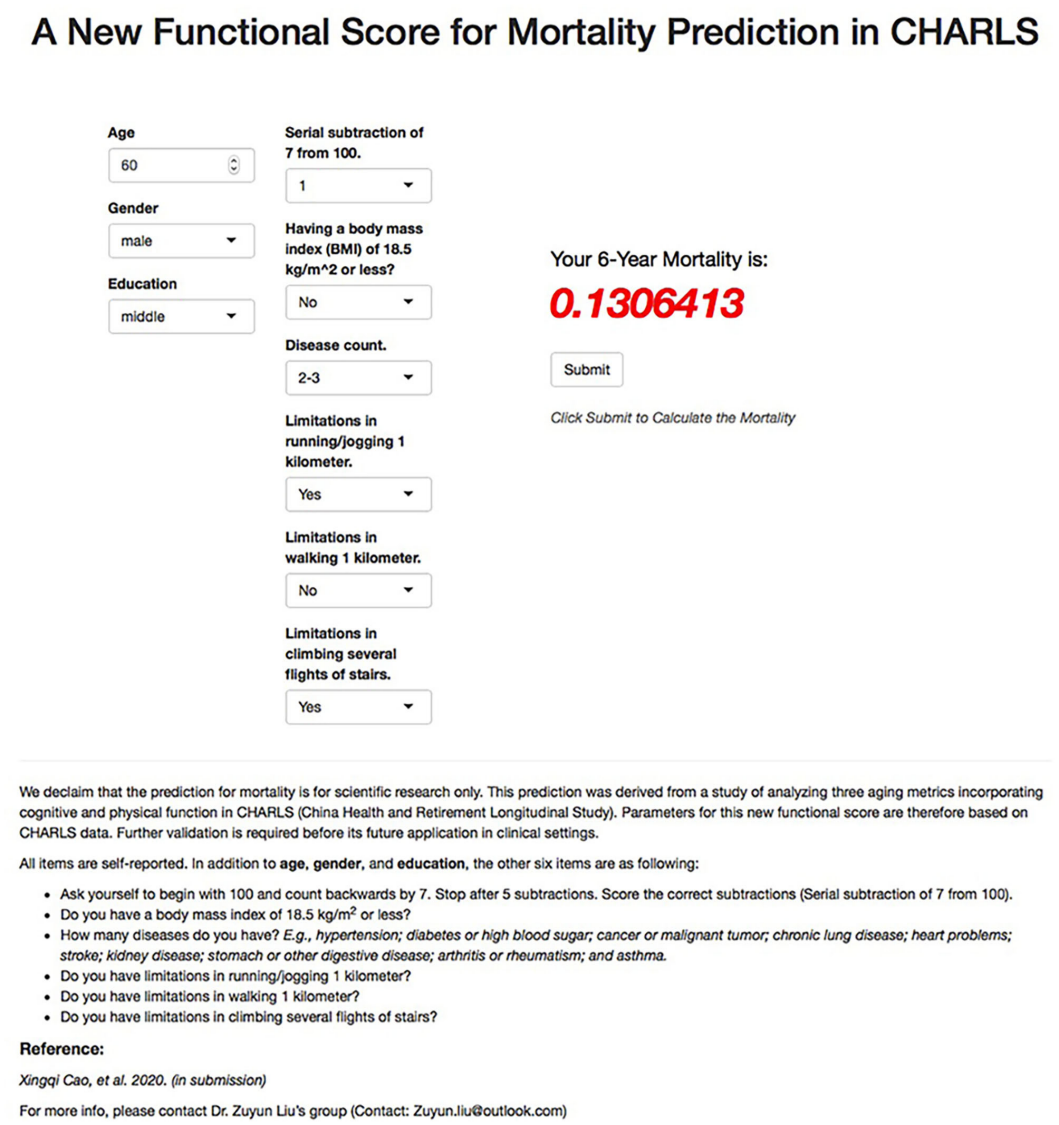
Illustration of all-cause mortality prediction for new functional score using the online tool for a 60-year-old Chinese person. CHARLS, China Health and Retirement Longitudinal Study.

To help the public use of all-cause mortality prediction using the newly developed simple functional score, we provided an illustrative online tool (https://zipoa.shinyapps.io/mortalityprediction) based on parameters from CHARLS. In addition to the six self-reported items that we included in the new functional score, we also included age, sex, and education. We included age and sex as they are extremely important for health and are generally known to each person. We included education because of the same reason, and more importantly, because it may have some effects on the cognition-related items in the functional score (i.e., serial subtraction of 7 from 100). The integration then allows the user to get to know about his/her 6-year all-cause mortality risk after answering all items. For example, suppose there was a 60-year-old Chinese male, who graduated from middle school, could only count backward to 93 from 100 when doing the serial subtraction, had a BMI of 25 kg/m^2^, had hypertension and diabetes now, was limited in running 1 km and climbing several flights but was perfect in walking. Then he could get his 6-year all-cause mortality prediction of 13.1% from our simple online tool (Figure 3).

In sensitivity analyses: (1) further adjustment for depression did not change results substantially (Supplementary Table 3); (2) the new functional score added predictive utility for all-cause mortality relative to the model including one existing metric (i.e., cognitive frailty, FI, or MCR), age, and sex in CHALRS (Supplementary Figure 1). For instance, relative to that of a model with cognitive frailty, age, and sex, adding the new functional score contributed significant increases in IDI (0.016, *P* < 0.001) and continuous NRI (0.264, *P* < 0.001) for predicting all-cause mortality.

## Discussion

As an illustrative example of how to balance feasibility and performance, we successfully developed and validated a new simple functional score using six self-reported items concerning cognitive and physical function in CHARLS. We demonstrated that this functional score was significantly associated with all-cause mortality risk. Moreover, its predictive utility was confirmed by increased AUC, IDI, and continuous NRI. The new functional score was well-replicable in another cohort of the Chinese population (i.e., RLAS). The findings suggest that the new functional score could assist in identifying vulnerable populations at risk in China, the largest developing country with a rapidly growing aging population.

There were many well-validated functional tools, such as BADL (3), IALD (4), SPPB (5), and function impairment screening tool (FIST) (25). However, these tools mainly focused on physical function and did not consider cognitive function. To the best of our knowledge, this is the first study to use self-reported items to develop a functional score incorporating cognitive and physical function. This new functional score comprised both cognitive (i.e., serial subtraction) and physical function domains (e.g., walking 1 km), representing two aspects of functional aging. Furthermore, six items contained in the new functional score were given different weights depending on their contribution to the heterogeneity of all-cause mortality risk. For instance, having limitations in climbing several flights of stairs was defined as 1 risk point, while having limitations in walking 1 km was defined as 5 risk points. This improved aging metrics previously developed [i.e., cognitive frailty (17), FI (18), and MCR (19)] which roughly give equal weight to each item of cognitive and physical function. In addition, the new functional score was developed on the basis of CHARLS, a nationally presentative cohort in China, and it was also validated in RLAS, another cohort of the aging population. These findings support the robustness of predicting all-cause mortality risk using the new functional score in the Chinese population.

The development of the new functional score has important implications for public and clinical work, not just because of its validity of all-cause mortality risk prediction, but also due to that the items included do not require time-consuming physical examinations. The new functional score has a great potential for early identification of functional aging, and thus, helps with effective interventions in time. Also, this new functional score could be used as an alternative endpoint to assess the effectiveness of clinical anti-aging interventions, without requiring long-time follow-up. For instance, the FI has been widely used as an indicator to evaluate the effect of anti-aging interventions, such as calorie restriction (e.g., metformin, rapamycin, and resveratrol) (26, 27). Furthermore, with the illustrative online tool, where each participant could calculate his/er 6-year all-cause mortality risk prediction, the new functional score has great feasibility and practicability.

The strength of this study is the study sample from two cohorts in China, including one national representative cohort and one cohort of the regional aging population. The development and validation of the new functional score were performed in two separate cohorts of the Chinese population, respectively, reinforcing our findings. However, there are also some limitations. First, one of the main limitations is the short follow-up period of our study (i.e., 6 years). Because of this, we are unable to examine the long-term effect of the new functional score on adverse health outcomes. Second, the new functional score and its predictive utility for all-cause mortality risk across various countries/regions may be different due to the influence of genetics, demographics, and economics on aging. Thus, more studies are required to repeat our analyses in various countries/regions and populations to test the validity of this new functional score. Finally, the predictive utility for all-cause mortality risk was relatively low in RLAS, which may be induced by either the short-term follow-up period or the exclusion of other important variables (e.g., BADL disability) that affect mortality when constructing this new functional score.

In summary, we developed a new functional score consisting of six self-reported cognitive and physical function items in the Chinese population, which was demonstrated to be able to predict all-cause mortality risk, showing good feasibility and performance. Furthermore, this functional score was validated in an independent cohort, strengthening its predictive utility across the Chinese population. Thus, the new simple functional score we developed has a great potential for early identification and prevention of functional aging in the older Chinese population. Nevertheless, it requires further validation in other countries/regions and populations.

## Data Availability Statement

The China Health and Retirement Longitudinal Study data (CHARLS) are available in the CHARLS website: http://charls.pku.edu.cn/en. The Rugao Longitudinal Ageing study (RLAS) data are available on request from the corresponding authors (Xiaofeng Wang and Zuyun Liu).

## Ethics Statement

The China Health and Retirement Longitudinal Study (CHARLS) was approved by the Biomedical Ethics Review Committee of Peking University, and all participants provide informed consent. The Rugao Longitudinal Ageing study (RLAS) was approved by the Human Ethics Committee of the School of Life Science at Fudan University, and all participants provide informed consent.

## Author Contributions

ZL: conceived and designed the study. XC and CC: performed the analysis and wrote the initial draft of the manuscript. LH, ZZ, JZ, EH, XL, SL, XW, YZ, and ZL: helped to interpret the results and edit the manuscript. EH, XW, YZ, and ZL: contributed to the critical revision of the manuscript for important intellectual contents. All authors read and approved the final version of the manuscript.

## Funding

This research was supported by a grant from the National Natural Science Foundation of China (82171584 and 72004201), the 2020 Irma and Paul Milstein Program for Senior Health project award (Milstein Medical Asian American Partnership Foundation), the Fundamental Research Funds for the Central Universities, a project from the Natural Science Foundation of Zhejiang Province (LQ21H260003), Shanghai Municipal Science and Technology Major Project (2017SHZDZX01), Key Laboratory of Intelligent Preventive Medicine of Zhejiang Province (2020E10004), and Zhejiang University Global Partnership Fund (188170-11103). The funders had no role in the study design; data collection, analysis, or interpretation; in the writing of the report; or in the decision to submit the article for publication.

## Conflict of Interest

The authors declare that the research was conducted in the absence of any commercial or financial relationships that could be construed as a potential conflict of interest.

## Publisher's Note

All claims expressed in this article are solely those of the authors and do not necessarily represent those of their affiliated organizations, or those of the publisher, the editors and the reviewers. Any product that may be evaluated in this article, or claim that may be made by its manufacturer, is not guaranteed or endorsed by the publisher.
