# Supplementary material for: Development and Validation of a New Simple Functional Score in the Older Chinese Population
Source: Front Public Health. 2022 Feb 24;10:813323. doi: 10.3389/fpubh.2022.813323 (PMC8907530; doi:10.3389/fpubh.2022.813323)
Supplement: Supplementary file 1 [file Data_Sheet_1.docx]

**Supplementary Table 1.** **The estimate of the risk for all-cause mortality of the new functional score in CHARLS.**

| Total points | Estimate of risk | Total points | Estimate of risk |
| --- | --- | --- | --- |
| 0 | 0.034 | 11 | 0.089 |
| 1 | 0.037 | 12 | 0.097 |
| 2 | 0.040 | 13 | 0.097 |
| 3 | 0.044 | 14 | 0.115 |
| 4 | 0.048 | 15 | 0.125 |
| 5 | 0.053 | 16 | 0.135 |
| 6 | 0.058 | 17 | 0.147 |
| 7 | 0.063 | 18 | 0.159 |
| 8 | 0.069 | 19 | 0.172 |
| 9 | 0.075 | 20 | 0.186 |
| 10 | 0.082 |  |  |

Notes: CHARLS, China Health and Retirement Longitudinal Study.

**Supplementary Table 2. Summary characteristics of the total participants in RLAS 2016.**

| **Characteristics** | Total | Male | Female |
| --- | --- | --- | --- |
| N | 1345 | 624 | 721 |
| Age, mean ± SD | 77.2 ± 3.9 | 77.1 ± 3.8 | 77.3 ± 3.9 |
| Education |  |  |  |
| Illiterate, N (%) | 703 (52.7) | 125 (20.2) | 578 (80.8) |
| Literate, N (%) | 632 (47.3) | 495 (79.8) | 137 (19.2) |
| BMI (kg/m^2^), mean ± SD | 23.7 ± 3.6 | 23.3 ± 3.2 | 24.1 ± 3.9 |
| Disease count^a^ |  |  |  |
| 0, N (%) | 366 (27.2) | 178 (28.5) | 188 (26.1) |
| 1, N (%) | 508 (37.8) | 249 (39.9) | 259 (35.9) |
| 2, N (%) | 293 (21.8) | 131 (21.0) | 162 (22.5) |
| 3, N (%) | 119 (8.9) | 43 (6.9) | 76 (10.6) |
| ≥4, N (%) | 59 (4.4) | 23 (3.7) | 36 (5.0) |
| Walking limitation, N (%) | 231 (17.2) | 58 (9.3) | 173 (24.0) |
| Climbing limitation, N (%) | 555 (41.3) | 182 (29.2) | 373 (51.7) |
| Serial subtraction score, mean ± SD | 2.5 ± 6.8 | 3.2 ± 1.3 | 1.8 ± 1.6 |
| HDS-R score, mean ± SD | 21.7 ± 6.8 | 25.6 ± 5.6 | 18.4 ± 5.9 |

Notes: RLAS, Rugao Longitudinal Ageing Study; SD, standard deviation; BMI, body mass index; HDS-R, Hierarchic Dementia Scale-Revised.

^a^Chronic diseases included hypertension, diabetes, cancer or malignant tumor, chronic lung disease, heart problems, stroke, kidney disease, stomach or other digestive diseases, arthritis or rheumatism, and asthma.

**Supplementary Table 3. Associations of the new functional score with all-cause mortality after additionally adjusting for depression in CHARLS.**

|  |  | No. of events/No. of participants | **Model 4** | |
| --- | --- | --- | --- | --- |
|  |  |  | OR (95% CI) | P value |
| New functional score | Per 1 score | 574/3929 | 1.10 (1.07, 1.13) | <0.001 |
|  |  |  |  |  |
| Quintiles | Q1 | 67/894 | Ref. | − |
|  | Q2 | 117/944 | 1.47 (1.06, 2.04) | 0.022 |
|  | Q3 | 85/657 | 1.63 (1.14, 2.33) | 0.007 |
|  | Q4 | 135/819 | 1.95 (1.39, 2.74) | <0.001 |
|  | Q5 | 170/615 | 3.17 (2.23, 4.50) | <0.001 |
|  | P for trend |  | — | <0.001 |

Notes: CHARLS, China Health and Retirement Longitudinal Study; OR, odds ratio; CI, confidence interval; Q1, the first quintile; Q2, the second quintile; Q3, the third quintile; Q4, the fourth quintile; Q5, the fifth quintile.

Model 4 adjusted for age, sex, residence, education, and depression (assessed by CESD-10).

**
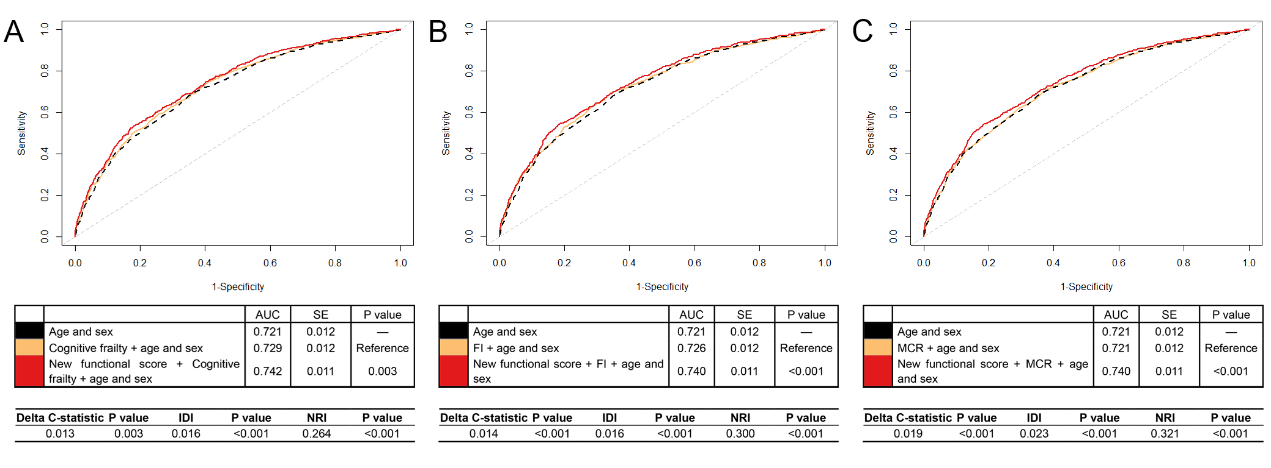
Supplementary Figure 1. The new functional score outperformed three existing metrics for all-cause mortality prediction in CHARLS.**

Notes: CHARLS, China Health and Retirement Longitudinal Study; AUC, area under the curve; SE, standard error; IDI, integrated discrimination improvement; NRI, net reclassification index; FI, frailty index; MCR, Motoric Cognitive Risk syndrome. We calculated the continuous NRI and IDI using the R package “PredictABEL”, in comparison to that of the model including one existing metric, age, and sex. NRI equals to x% means that compared with persons without outcome, persons with outcome were almost x% more likely to move up a category than down. IDI equals to x% means that the difference in average predicted risks between the persons with and without the outcome increased by x% in the updated model. A shows that the new functional score outperformed cognitive frailty in predicting all-cause mortality. B shows that the new functional score outperformed FI in predicting all-cause mortality. C shows that the new functional score outperformed MCR in predicting all-cause mortality. Results of the models including one existing metric (i.e., cognitive frailty, FI, or MCR), age, and sex in CHARLS were previously reported in our preprint (doi: https://doi.org/10.1101/2021.05.14.21257213).
